# Network pharmacology and molecular docking analysis on the mechanism of Baihe Zhimu decoction in the treatment of postpartum depression

**DOI:** 10.1097/MD.0000000000029323

**Published:** 2022-10-28

**Authors:** Qiong Zhao, Wengu Pan, Hongshuo Shi, Fanghua Qi, Yuan Liu, Tiantian Yang, Hao Si, Guomin Si

**Affiliations:** a School of Traditional Chinese Medicine, Shandong University of Traditional Chinese Medicine, Jinan, China; b Department of Traditional Chinese Medicine, Shandong Provincial Hospital Affiliated to Shandong University, Jinan, China; c Department of Kidney transplantation, The second hospital of Shandong University, Jinan, China; d Ai Kunwei Pharmaceutical Technology Co, Ltd, Shanghai, China.

**Keywords:** Baihe Zhimu decoction, molecular docking, network pharmacology, postpartum depression, signaling pathway

## Abstract

Baihe Zhimu decoction (BZD) has significant antidepressant properties and is widely used to treat mental diseases. However, the multitarget mechanism of BZD in postpartum depression (PPD) remains to be elucidated. Therefore, the aim of this study was to explore the molecular mechanisms of BDZ in treating PPD using network pharmacology and molecular docking. Active components and their target proteins were screened from the traditional Chinese Medicine Systems Pharmacology Database and Analysis Platform (TCMSP). The PPD-related targets were obtained from the OMIM, CTD, and GeneCards databases. After overlap, the targets of BZD against PPD were collected. Protein–protein interaction (PPI) network and core target analyses were conducted using the STRING network platform and Cytoscape software. Moreover, molecular docking methods were used to confirm the high affinity between BZD and targets. Finally, the DAVID online tool was used to perform gene ontology (GO) and Kyoto Encyclopedia of Genes and Genomes (KEGG) pathway enrichment analysis of overlapping targets. The TCMSP database showed that BZD contained 23 active ingredients in PPD. KEGG analysis showed that overlapping genes were mainly enriched in HIF-1, dopaminergic synapses, estrogen, and serotonergic synaptic signalling pathways. Combining the PPI network and KEGG enrichment analysis, we found that ESR1, MAOA, NR3C1, VEGFA, and mTOR were the key targets of PPD. In addition, molecular docking confirmed the high affinity between BZD and the PPD target. Verified by a network pharmacology approach based on data mining and molecular docking methods, the multi-target drug BZD may serve as a promising therapeutic candidate for PPD, but further in vivo/in vitro experiments are needed.

## 1. Introduction

Postpartum depression (PPD) can be defined as non-psychotic depression occurring within a year of childbirth, characterized by low mood, unusual thoughts, feelings of guilt, unexplained anxiety, worthlessness, and other depressive symptoms.^[1]^ The rate of PPD among women may be as high as 15% and result in a high death rate from self-harm. The rising numbers could make matters worse.^[2–5]^ Thus, PPD is a significant public health problem.

However, the aetiology of PPD remains unclear. Various factors are associated with PPD, such as low socioeconomic status, prenatal depression, cultural factors, medication use, excessive stress, and anxiety.^[6,7]^ Hormonal, genetic, and psychological effects can all lead to PPD, resulting in a series of physical, mental, and behavioral changes.^[8]^ There is no consensus on drugs for the treatment of PPD, although there are reports of the use of antidepressants and antipsychotics in severe cases.^[9]^ Big data fails to confirm whether antidepressant treatment produces adverse effects on breastfed infants.^[10,11]^

Traditional Chinese medicine (TCM) is an oriental traditional medicine that is characterized by a holistic concept. It was the main medical method used in ancient China, with thousands of years of accumulated practical experience.^[12]^ In ancient China, TCM was used to treat many mental illnesses, such as restlessness, lily disease, and depression. Reports show that many TCMs, such as Baihe Zhimu decoction (BZD), Chaihu decoction, and Zhizichi decoction Xiaoyao san, are used to treat PPD.

BZD is a classic traditional Chinese medicine prescription, which was first recorded in Treatise on Febrile and Miscellaneous Diseases (200–210 ad). Consisting of two herbs, Lilii Bulbus (Baihe BH) and Anemarrhenae Rhizoma (Zhimu ZM), BZD has become a classic Chinese medicine formulation for treating depression, insomnia, anxiety and other mental and neurological diseases.^[13]^ Some studies have shown that BZD can increase 5-hydroxytryptamine, norepinephrine, and dopamine levels and improve the behavioral indicators of depression.^[14–17]^ Clinical data indicate that Morita therapy combined with BZD can improve the symptoms, quality of life, and ability of daily living for first-episode depression, which may be related to the decrease in serum BDNF and an increase in DOPAC levels.^[18]^ However, the potential mechanisms underlying the treatment of PPD are not fully understood.

Network pharmacology is an emerging method that analyses the components, targets, diseases, and related pathways of traditional Chinese medicine. It combines pharmacology, molecular biology, electronic technology, and bioinformatics and has great advantages for studying the complex components, targets, and pathways of traditional Chinese medicine prescriptions.^[19–21]^

**Figure 1.**
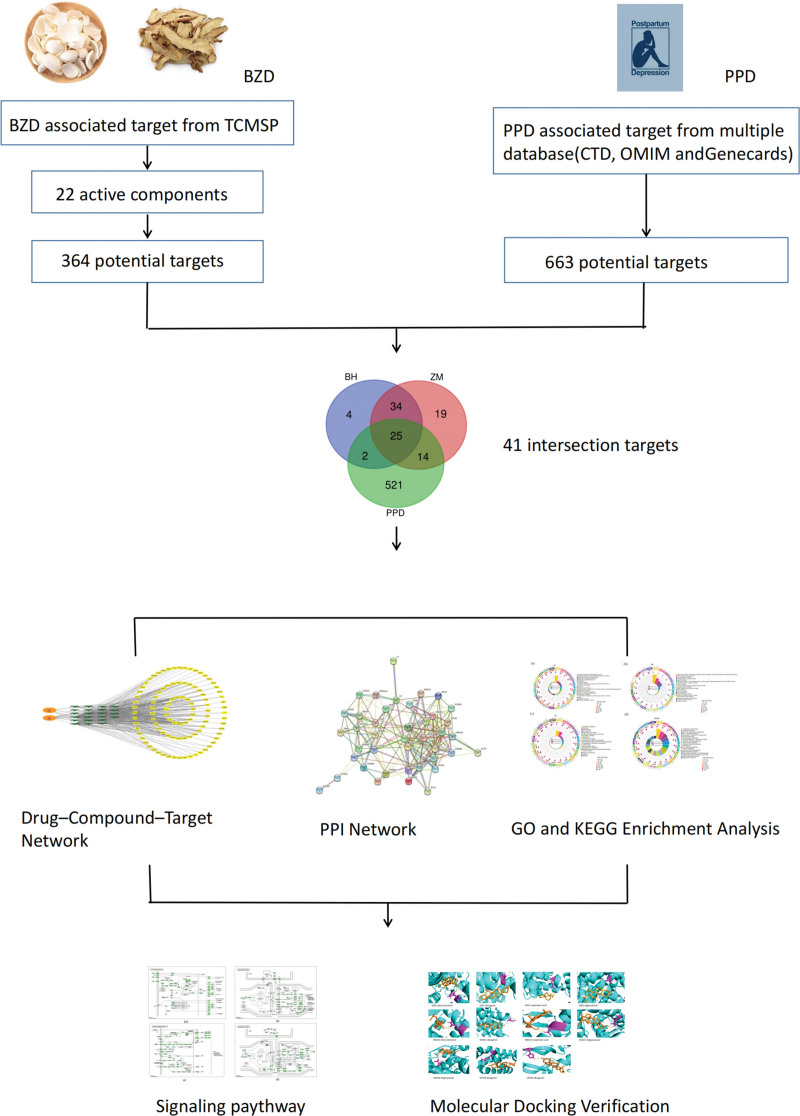
Workflow of the study design.

Therefore, this research aims to apply network pharmacology to identify the active ingredients and targets of BZD and to discover the common targets and possible signaling pathways of BZD in the treatment of PPD. The corresponding workflow is shown in Figure 1.

## 2. Materials and Methods

### 2.1. Chemical ingredient acquisition and processing

The components of the two herbs were found in the pharmacology of Traditional Chinese Medicine Systems Pharmacology (TCMSP, https://tcmspw.com/tcmsp.php)^[22]^ and then screened by integrating oral bioavailability (OB ≥ 30%) and drug-likeness (DL ≥ 0.18).^[23]^ Next, each target of the active ingredient was obtained from this website. The aggregated target was input into UniProt (https://www.uniprot.org/) to obtain information, such as gene symbols and gene IDs.^[24,25]^

### 2.2. Related targets of PPD

PPD targets were obtained from the Online Mendelian Inheritance in Man (OMIM, https://www.omim.org/),^[26]^ Comparative Toxicogenomics Database (CTD, http://ctd.mdibl.org/),^[27]^ and GeneCards (https://www.genecards.org/).^[28,29]^ We searched for “postpartum depression” in the GeneCards, CTD, and OMIM databases. Finally, all targets were unified as gene names on UniProt.

### 2.3. Construction of drug–compound–target genes network

We input the information obtained above of BZD-related drugs, components, and targets into Cytoscape 3.7.2 software to construct a visualized network diagram of the drug-component-target. The graph depicts drugs, ingredients and targets as nodes and the lines connecting them as edges.

### 2.4. Venn diagram of targets between drugs and disease

The targets of BZD and PPD-related target gene lists were input into Venny 2.1.0 (http://bioinformatics.psb.ugent.be/webtools/Venn/) to obtain their intersection with a Venn diagram.

### 2.5. Protein–protein interaction data

A protein–protein interaction (PPI) network for disease and drug mapping targets was constructed using the STRING database (https://string-db.org/, version 10.5). According to the corresponding calculation method, the species was set to “Homo sapiens,” and the confidence score was set to >0.4.^[30]^

### 2.6. Gene ontology and pathway enrichment analysis

The Database for Annotation, Visualization, and Integrated Discovery (DAVID; http://david.abcc.ncifcrf.gov/)^[31,32]^ was used for gene ontology (GO) and Kyoto Encyclopedia of Genes and Genomes (KEGG) pathway enrichment analyses.

### 2.7. Molecular docking

Based on the results of the GO and KEGG pathway analyses, we selected the key protein receptor and ligand associated with the protein receptor. PubChem (https://pubchem.ncbi.nlm.gov/)^[33]^ was used to obtain the 2D chemical structures of the small-molecule ligands. ChemOffice software was used to construct the 3D chemical structures of small molecular ligands,^[34]^ and the 3D chemical structures of protein receptors were acquired from PDB (http://www.rcsb.org/).^[35]^ After the molecular ligands and water molecules of the protein receptors were removed using PyMol 2.4.0 software (https://pymol.org.),^[36]^ the format of the protein receptors and small-molecule ligands was transformed into pdbqt format.^[37]^ AutoDock Vina was used for molecular docking.^[38]^ Based on the binding energy value, the lowest binding energy value was selected as the docking affinity. Finally, PyMol software was used to visualize the 3D structures of the molecular ligand and protein receptor bonding.

## 3. Results

### 3.1. Chemical components of BZD

The active compounds of BZD were retrieved from TCMSP. There were 165 related components in BZD; Baihe contained 84 (50.9%), and Zhimu had 81 (49.1%) components. The values of OB and DL (OB ≥ 30% and DL ≥ 0.18) were used to screen potential active compounds from Baihe and Zhimu, and 22 active compounds met the screening standards. The properties of the compounds are listed in Table 1. The 364 targets and the corresponding Uniprot IDs are listed in Table 2.

**Table 1:** Compounds of Baihe Zhimu decoction.

Mol ID	Molecule name	OB (%)	DL	Herbs
MOL009471	26-O-ß-D-glucopyranosyl-3ß,26-dihydroxy-cholestan-16,22-dioxo-3-O-a-L-rhamnopyranosyl-(1?2)-ß-D-glucopyranoside_qt	32.43	0.8	BH
MOL009465	26-O-ß-D-glucopyranosyl-3ß,26-dihydroxy-5-cholesten-16,22-dioxo-3-O-a-L-rhamnopyranosyl-(1?2)-ß-D-glucopyranoside_qt	35.11	0.81	BH
MOL009458	3-Demethylcolchicine	39.34	0.57	BH
MOL009449	26-O-beta-D-Glucopyranosyl-3beta,26-dihydroxy-choleslen-16,22-dioxo-3-O-alpha-L-rhamnopyranosyl-(1-2)-beta-D-glucopyranoside_qt	32.43	0.8	BH
MOL002039	Isopimaric acid	36.2	0.28	BH
MOL000449	Stigmasterol	43.83	0.76	BH
MOL000358	beta-Sitosterol	36.91	0.75	BH
MOL001677	Asperglaucide	58.02	0.52	ZM
MOL001944	Marmesin	50.28	0.18	ZM
MOL003773	Mangiferolic acid	36.16	0.84	ZM
MOL000422	Kaempferol	41.88	0.24	ZM
MOL004373	Anhydroicaritin	45.41	0.44	ZM
MOL004489	Anemarsaponin F_qt	60.06	0.79	ZM
MOL004492	Chrysanthemaxanthin	38.72	0.58	ZM
MOL004497	Hippeastrine	51.65	0.62	ZM
MOL004514	Timosaponin B III_qt	35.26	0.87	ZM
MOL000449	Stigmasterol	43.83	0.76	ZM
MOL004528	Icariin I	41.58	0.61	ZM
MOL004540	Anemarsaponin C_qt	35.5	0.87	ZM
MOL004542	Anemarsaponin E_qt	30.67	0.86	ZM
MOL000483	(Z)-3-(4-hydroxy-3-methoxy-phenyl)-N-[2-(4-hydroxyphenyl)ethyl]acrylamide	118.35	0.26	ZM
MOL000546	Diosgenin	80.88	0.81	ZM
MOL000631	Coumaroyltyramine	112.9	0.2	ZM

**Table 2:** Information of 364 targets of Baihe Zhimu decoction.

No.	Target	UniprotID	No.	Target	UniprotID	No.	Target	UniprotID	No.	Target	UniprotID
1	ESR1	P03372	92	PTGS2	P35354	183	F2	P00734	274	ESR1	P03372
2	AR	P10275	93	CA2	P00918	184	CHRM1	P11229	275	AR	P10275
3	PGR	P06401	94	GABRA2	P47869	185	AR	P10275	276	ADRB1	P08588
4	NR3C1	P04150	95	CHRM4	P08173	186	PPARG	P37231	277	SCN5A	Q14524
5	ESR1	P03372	96	ACHE	P22303	187	NOS3	P29474	278	PPARG	P37231
6	AR	P10275	97	PDE3A	Q14432	188	CA2	P00918	279	PTGS2	P35354
7	NR3C2	P08235	98	HTR2A	P28223	189	F7	P08709	280	NOS3	P29474
8	NR3C1	P04150	99	GABRA5	P31644	190	GABRA2	P47869	281	ADRA2A	P08913
9	NOS2	P35228	100	ADRA1A	P35348	191	ACHE	P22303	282	CA2	P00918
10	PTGS1	P23219	101	GABRA3	P34903	192	SLC6A2	P23975	283	RXRA	P19793
11	F2	P00734	102	PGR	P06401	193	PGR	P06401	284	ACHE	P22303
12	KCNH2	Q12809	103	CHRM2	P08172	194	CHRM2	P08172	285	HTR2A	P28223
13	ESR1	P03372	104	ADRA1B	P35368	195	ADRA1B	P35368	286	SLC6A2	P23975
14	AR	P10275	105	PTPN1	P18031	196	PTPN1	P18031	287	ADRA1A	P35348
15	PTGS2	P35354	106	ADRB2	P07550	197	GABRA1	P14867	288	GABRA3	P34903
16	DPP4	P27487	107	CHRNA2	Q15822	198	DPP4	P27487	289	PGR	P06401
17	CHEK1	O14757	108	SLC6A4	P31645	199	MAPK14	Q16539	290	CHRM2	P08172
18	ESR1	P03372	109	OPRM1	P35372	200	GSK3B	P49841	291	ADRA1B	P35368
19	AR	P10275	110	ESR2	Q92731	201	CDK2	P24941	292	SLC6A3	Q01959
20	NR3C2	P08235	111	NR3C1	P04150	202	PIK3CG	P48736	293	NR3C2	P08235
21	NR3C1	P04150	112	GABRA1	P14867	203	LACTB	P83111	294	ADRB2	P07550
22	NOS2	P35228	113	DPP4	P27487	204	CHEK1	O14757	295	AKR1B1	P15121
23	F2	P00734	114	MAPK14	Q16539	205	PRKACA	P17612	296	NR3C1	P04150
24	ESR1	P03372	115	GSK3B	P49841	206	PRSS1	P07477	297	GABRA1	P14867
25	AR	P10275	116	CDK2	P24941	207	PIM1	P11309	298	DPP4	P27487
26	PTGS2	P35354	117	PIK3CG	P48736	208	CCNA2	P20248	299	PLAU	P00749
27	RXRA	P19793	118	LACTB	P83111	209	NCOA2	Q15596	300	CDK2	P24941
28	ACHE	P22303	119	CHRNA7	P36544	210	NOS2	P35228	301	LACTB	P83111
29	PGR	P06401	120	CHEK1	O14757	211	PTGS1	P23219	302	LTA4H	P09960
30	NR3C1	P04150	121	PRKACA	P17612	212	CHRM3	P20309	303	MAOB	P27338
31	NCOA2	Q15596	122	PRSS1	P07477	213	F2	P00734	304	MAOA	P21397
32	NCOA1	Q15788	123	PIM1	P11309	214	KCNH2	Q12809	305	CHRNA7	P36544
33	NOS2	P35228	124	CCNA2	P20248	215	CHRM1	P11229	306	PRKACA	P17612
34	PTGS1	P23219	125	NCOA2	Q15596	216	ESR1	P03372	307	ADH1C	P00326
35	CHRM3	P20309	126	NOS2	P35228	217	AR	P10275	308	IGHG1	P01857
36	F2	P00734	127	F2	P00734	218	SCN5A	Q14524	309	CTRB1	P17538
37	CHRM1	P11229	128	KCNH2	Q12809	219	PPARG	P37231	310	PRSS1	P07477
38	ESR1	P03372	129	ESR1	P03372	220	F10	P00742	311	NCOA2	Q15596
39	AR	P10275	130	PPARG	P37231	221	CHRM5	P08912	312	NCOA1	Q15788
40	ADRB1	P08588	131	F10	P00742	222	PTGS2	P35354	313	ESR1	P03372
41	SCN5A	Q14524	132	PTGS2	P35354	223	NOS3	P29474	314	AR	P10275
42	PPARG	P37231	133	PRSS1	P07477	224	CA2	P00918	315	PGR	P06401
43	PTGS2	P35354	134	NOS2	P35228	225	F7	P08709	316	NR3C2	P08235
44	NOS3	P29474	135	PTGS1	P23219	226	KDR	P35968	317	NR3C1	P04150
45	ADRA2A	P08913	136	F2	P00734	227	RXRA	P19793	318	AR	P10275
46	CA2	P00918	137	CHRM1	P11229	228	ACHE	P22303	319	PTGS1	P23219
47	RXRA	P19793	138	ESR1	P03372	229	ADRA1B	P35368	320	F2	P00734
48	ACHE	P22303	139	AR	P10275	230	PTPN1	P18031	321	ESR1	P03372
49	HTR2A	P28223	140	PTGS2	P35354	231	ADRB2	P07550	322	AR	P10275
50	SLC6A2	P23975	141	CA2	P00918	232	ESR2	Q92731	323	PPARG	P37231
51	ADRA1A	P35348	142	RXRA	P19793	233	DPP4	P27487	324	PTGS2	P35354
52	GABRA3	P34903	143	CHRM2	P08172	234	MAPK14	Q16539	325	PDE3A	Q14432
53	PGR	P06401	144	PTPN1	P18031	235	GSK3B	P49841	326	ADRA1B	P35368
54	CHRM2	P08172	145	ADRB2	P07550	236	CDK2	P24941	327	ADRB2	P07550
55	ADRA1B	P35368	146	SLC6A4	P31645	237	CHEK1	O14757	328	DPP4	P27487
56	SLC6A3	Q01959	147	DPP4	P27487	238	RXRB	P28702	329	MAPK14	Q16539
57	NR3C2	P08235	148	MAPK14	Q16539	239	IGHG1	P01857	330	CDK2	P24941
58	ADRB2	P07550	149	CDK2	P24941	240	PRSS1	P07477	331	LTA4H	P09960
59	AKR1B1	P15121	150	PIK3CG	P48736	241	PIM1	P11309	332	CHEK1	O14757
60	NR3C1	P04150	151	LACTB	P83111	242	CCNA2	P20248	333	PRSS1	P07477
61	GABRA1	P14867	152	LTA4H	P09960	243	NCOA2	Q15596	334	PIM1	P11309
62	DPP4	P27487	153	CHEK1	O14757	244	NCOA1	Q15788	335	ESR1	P03372
63	PLAU	P00749	154	PRKACA	P17612	245	KCNMA1	Q12791	336	PTGS2	P35354
64	CDK2	P24941	155	PIM1	P11309	246	ESR1	P03372	337	PLA2G4A	P47712
65	LACTB	P83111	156	ESR1	P03372	247	AR	P10275	338	FASN	P49327
66	LTA4H	P09960	157	AR	P10275	248	NR3C1	P04150	339	MTOR	P42345
67	MAOB	P27338	158	NOS2	P35228	249	F2	P00734	340	SOD1	P00441
68	MAOA	P21397	159	INSR	P06213	250	CHRM3	P20309	341	TP63	Q9H3D4
69	CHRNA7	P36544	160	ESR1	P03372	251	CHRM1	P11229	342	CAT	P04040
70	PRKACA	P17612	161	BCL2	P10415	252	ESR1	P03372	343	VEGFA	P15692
71	ADH1C	P00326	162	ALOX5	P09917	253	AR	P10275	344	AR	P10275
72	IGHG1	P01857	163	PTGS2	P35354	254	SCN5A	Q14524	345	PGR	P06401
73	CTRB1	P17538	164	AKR1C3	P42330	255	OPRD1	P41143	346	NR3C2	P08235
74	PRSS1	P07477	165	TNFSF15	O95150	256	ADRA1B	P35368	347	NR3C1	P04150
75	NCOA2	Q15596	166	ESR2	Q92731	257	OPRM1	P35372	348	PTGS1	P23219
76	NCOA1	Q15788	167	MMP1	P03956	258	GABRA1	P14867	349	F2	P00734
77	BCL2	P10415	168	JUN	P05412	259	DPP4	P27487	350	CHRM1	P11229
78	PON1	P27169	169	SELE	P16581	260	GSK3B	P49841	351	ESR1	P03372
79	JUN	P05412	170	CDK1	P06493	261	CDK2	P24941	352	ADRB1	P08588
80	MAP2	P11137	171	VCAM1	P19320	262	PIK3CG	P48736	353	PPARG	P37231
81	NOS2	P35228	172	XDH	P47989	263	CHRNA7	P36544	354	PTGS2	P35354
82	PTGS1	P23219	173	CYP3A4	P08684	264	CCNA2	P20248	355	ADRB2	P07550
83	DRD1	P21728	174	MAPK8	P45983	265	ESR1	P03372	356	DPP4	P27487
84	CHRM3	P20309	175	CYP1A2	P05177	266	AR	P10275	357	MAPK14	Q16539
85	F2	P00734	176	GSTP1	P09211	267	PGR	P06401	358	GSK3B	P49841
86	KCNH2	Q12809	177	HMOX1	P09601	268	NR3C1	P04150	359	CDK2	P24941
87	CHRM1	P11229	178	GSTM1	P09488	269	NOS2	P35228	360	LTA4H	P09960
88	ESR1	P03372	179	AHR	P35869	270	PTGS1	P23219	361	MAOB	P27338
89	AR	P10275	180	GSTM2	P28161	271	CHRM3	P20309	362	PRKACA	P17612
90	SCN5A	Q14524	181	PPP3CA	Q08209	272	F2	P00734	363	PRSS1	P07477
91	PPARG	P37231	182	PTGS1	P23219	273	CHRM1	P11229	364	PKIA	P61925

### 3.2. Screening targets of PPD

We searched for PPD targets in several publicly available databases: CTD, OMIM, and GeneCards. After deleting duplicate values, we finally obtained 663 disease targets.

### 3.3. Drug–compound–target network

**Figure 2.**
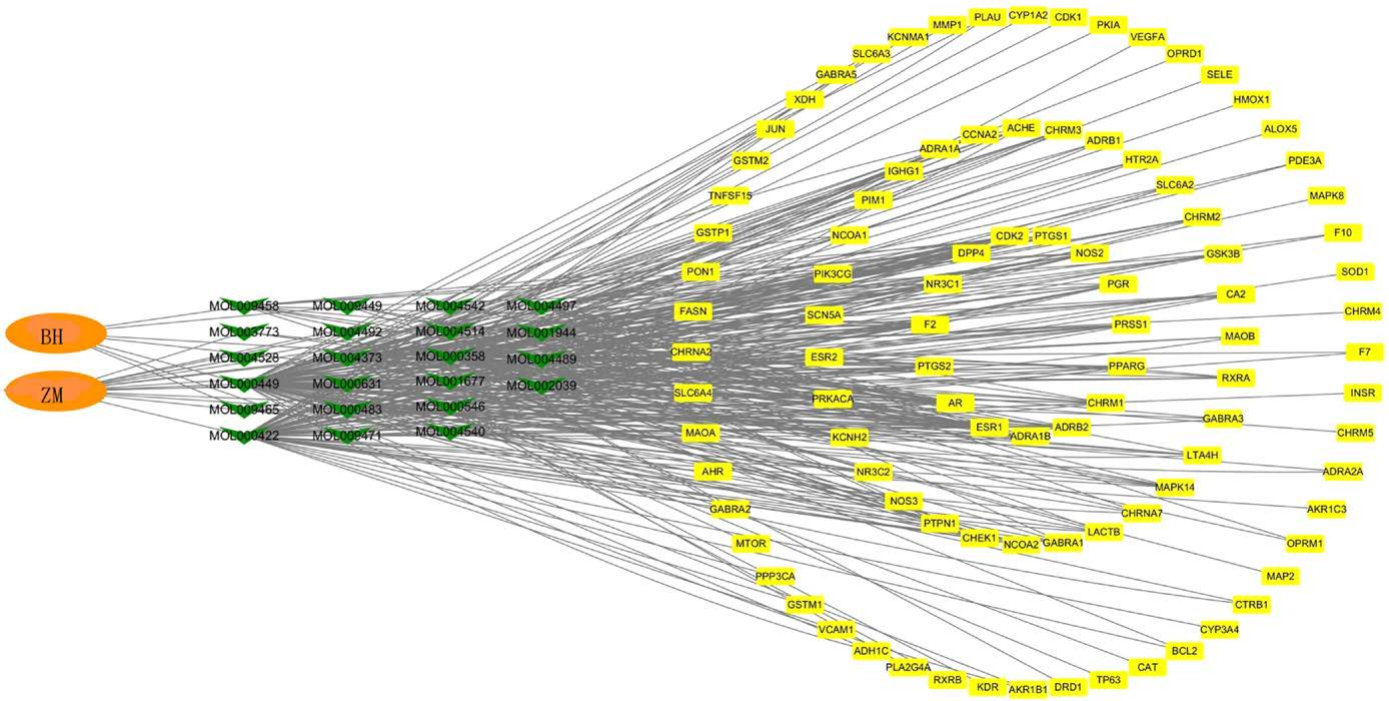
Drug-Compound-Target network. The orange color indicates the drugs; the green color indicates the chemical composition; the yellow color indicates the targets.

The drugs, components, and targets of BZD were drawn into a drug-component-target network diagram using the Cytoscape software to show the relationship between them more intuitively (Fig. 2). In this figure, orange represents two drugs, green rectangle represents 22 active ingredients, and yellow circle represents 364 target genes. Among the three circles, the degree value of the inner circle is higher than that of the outer circle, which has a higher correlation. In the drug-compound-target network, according to the degree value, the top 10 targets were ESR1, AR, F2, PTGS2, NR3C1, DPP4, PGR, NOS2, PTGS1, and CDK2.

### 3.4. Target protein cross-validation

**Figure 3.**
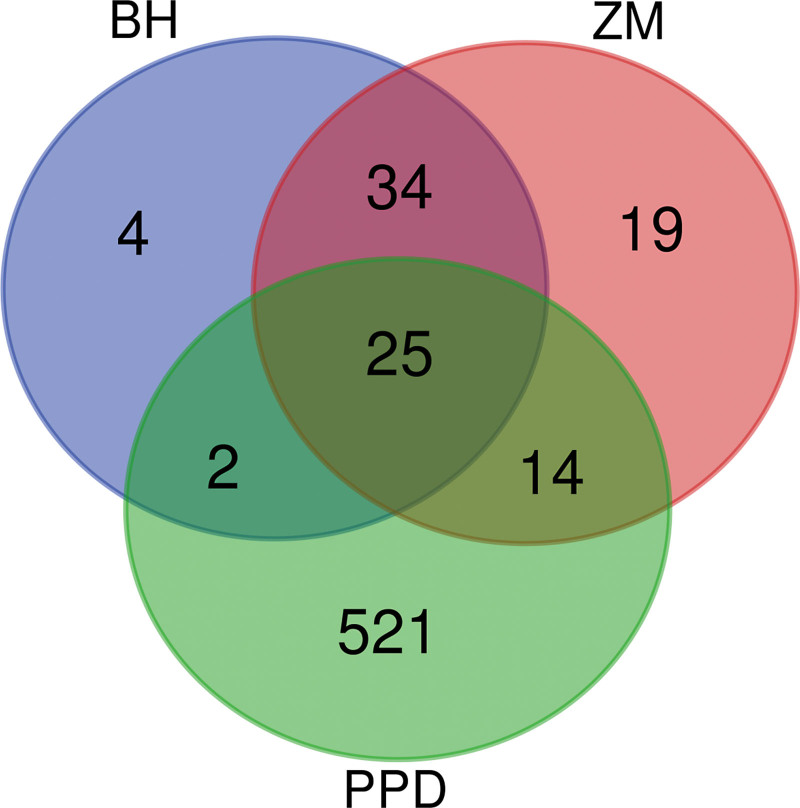
Drug-disease target Venn diagram of Baihe Zhimu decoction.

Figure 3 shows the set relationship between the targets of BZD and PPD. We input the 364 targets of BZD and 663 targets of PPD into the Venny2.1 online software mapping tool platform to draw the Venn diagram. Among them, 41 target proteins were targets of BZD acting on PPD, and 25 were common targets of Baihe, Zhimu, and PPD.

### 3.5. PPI network data

**Figure 4.**
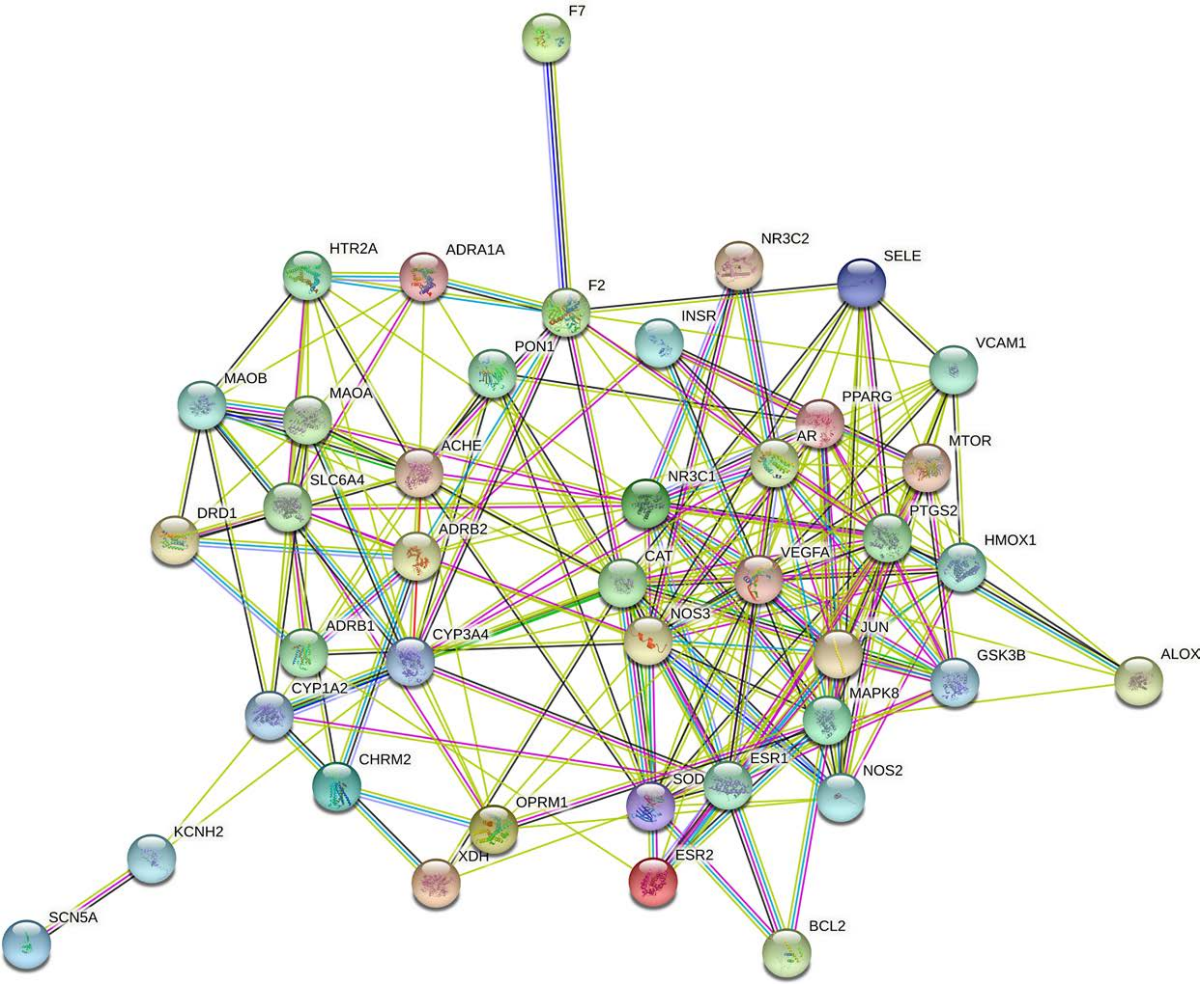
PPI network of core targets.

Figure 4 shows the PPI diagram of the 41 intersecting target proteins drawn using STRING. Red, evidence of fusion; green, evidence of proximity; yellow, evidence of text mining; light blue, evidence of database; blue, evidence of coexistence; black, evidence of co-expression; purple, evidence of experiment.

### 3.6. GO and pathway enrichment analysis

**Figure 5.**
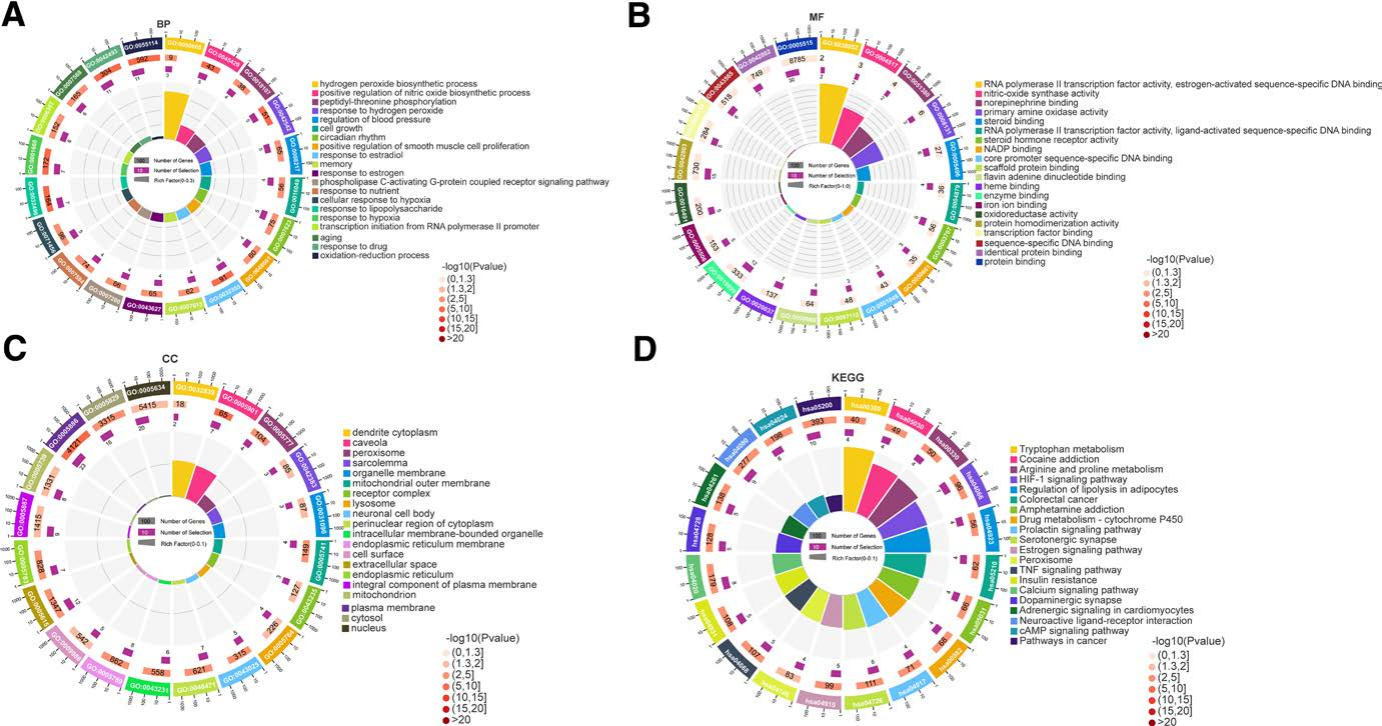
GO enrichment analysis of key targets and KEGG enrichment analysis. (A) The first 20 significant *P* values of BP) (B) The first 20 significant *P* values of CC; (C) The first 20 significant *P* values of MF; (D) The first 20 significant *P* values of KEGG pathways. BP = biological process, CC = cellular component; MF = molecular function.

**Figure 6.**
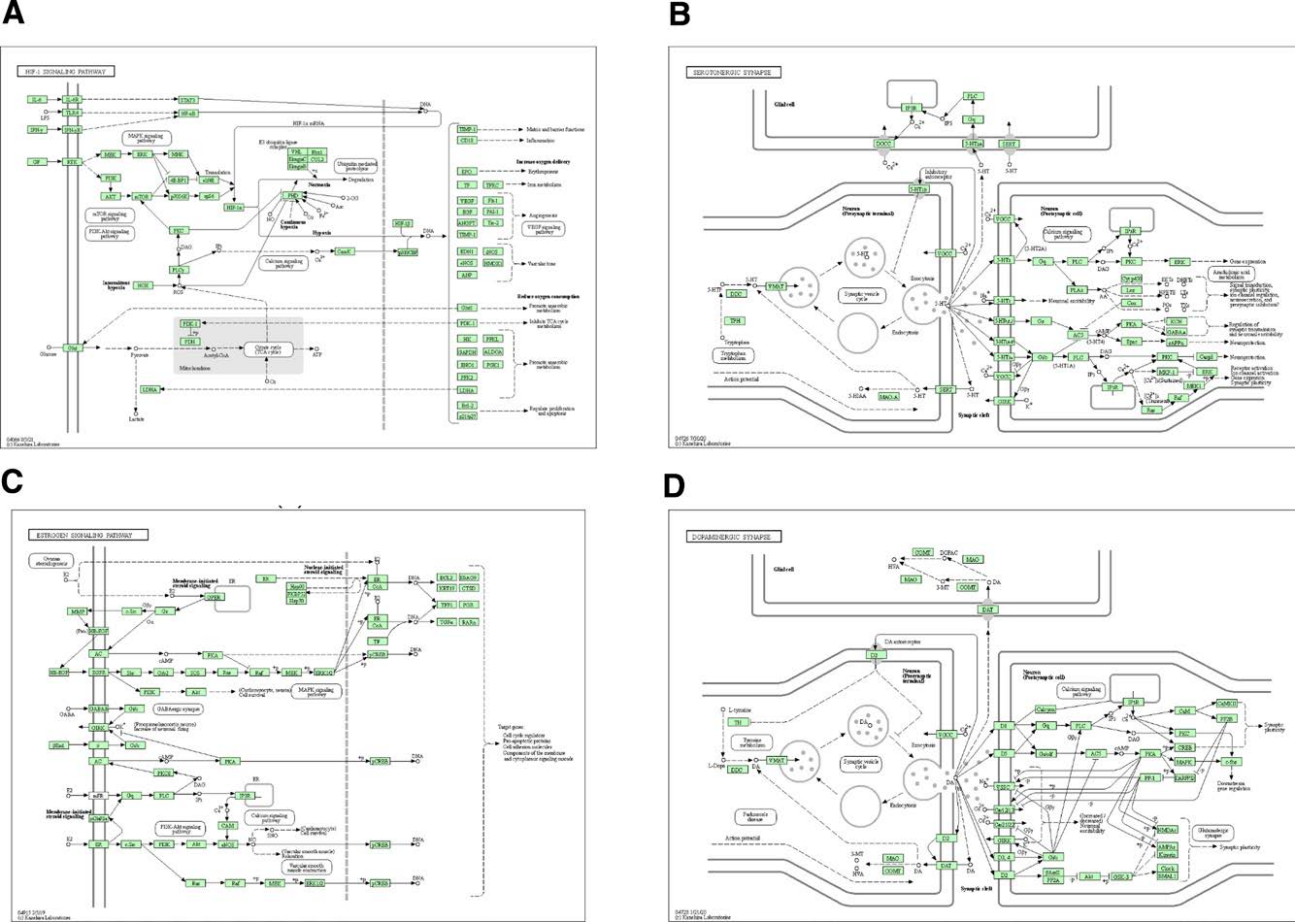
The ralated signaling pathways (obtained from KEGG database). (A) HIF-1 signaling pathway Serotonergic synapse; (B) Serotonergic synapse; (c) Estrogen signaling pathway; (D) Dopaminergic synapse.

The KEGG analysis and GO enrichment analysis of biological process (BP), molecular function (MF), and cell component (CC) obtained by DAVID 6.8 for 41 selected target genes are shown in Figure 5, which clarifies the multiple mechanisms of BZD in treating PPD. There is information on the number of genes, selections, and rich factors. P in the figure represents the significance of enrichment; the redder the color, the higher the significance. The five most affected BPs (*P* < .01) were response to drugs (GO:0042493), oxidation-reduction process (GO:0055114), response to lipopolysaccharide (GO:0032496), response to estradiol (GO:0032355), and response to hypoxia (GO:0001666) (Fig. 5A). The main CC terms (*P* < .01) were caveola (GO:0005901), plasma membrane (GO:0005886), extracellular space (GO:0005615), peroxisome (GO:0005777), and perinuclear region of the cytoplasm (GO:0048471) (Fig. 5B). The five most common MF terms (*P* < .01) were enzyme binding (GO:0019899), protein homodimerization activity (GO:0042803), steroid binding (GO:0005496), steroid hormone receptor activity (GO:0003707), and heme binding (GO:0020037) (Fig. 5C). The 41 proteins further resulted in 45 KEGG pathways. The top 20 pathways and their related genes are listed in Table 3. The relevant reference values are shown in Figure 5D. According to Figure 6, HIF-1, neuroactive ligand-receptor interaction, dopaminergic synapse, estrogen, and serotonergic synapse signaling pathways, which were filtered out as prominent and conspicuous enriched pathways, contributed significantly to the PPD response.

**Table 3:** Top 20 signaling pathways with related genes.

Term	Pathway	Genes
hsa04066	HIF-1 signaling pathway	NOS2, NOS3, INSR, BCL2, HMOX1, MTOR, VEGFA
hsa04020	Calcium signaling pathway	CHRM2, NOS2, NOS3, ADRB1, DRD1, ADRB2, HTR2A, ADRA1A
hsa04080	Neuroactive ligand-receptor interaction	CHRM2, ADRB1, DRD1, ADRB2, HTR2A, OPRM1, F2, NR3C1, ADRA1A
hsa05200	Pathways in cancer	GSK3B, AR, JUN, MAPK8, NOS2, BCL2, PPARG, PTGS2, MTOR, VEGFA
hsa04726	Serotonergic synapse	MAOB, MAOA, ALOX5, HTR2A, PTGS2, SLC6A4
hsa00380	Tryptophan metabolism	MAOB, MAOA, CYP1A2, CAT
hsa04915	Estrogen signaling pathway	JUN, NOS3, OPRM1, ESR1, ESR2
hsa05030	Cocaine addiction	JUN, MAOB, MAOA, DRD1
hsa00330	Arginine and proline metabolism	MAOB, NOS2, MAOA, NOS3
hsa04668	TNF signaling pathway	JUN, MAPK8, VCAM1, PTGS2, SELE
hsa04931	Insulin resistance	GSK3B, MAPK8, NOS3, INSR, MTOR
hsa04923	Regulation of lipolysis in adipocytes	INSR, ADRB1, ADRB2, PTGS2
hsa04024	cAMP signaling pathway	CHRM2, JUN, MAPK8, ADRB1, DRD1, ADRB2
hsa05210	Colorectal cancer	GSK3B, JUN, MAPK8, BCL2
hsa04728	Dopaminergic synapse	GSK3B, MAPK8, MAOB, MAOA, DRD1
hsa05031	Amphetamine addiction	JUN, MAOB, MAOA, DRD1
hsa00982	Drug metabolism - cytochrome P450	MAOB, MAOA, CYP1A2, CYP3A4
hsa04261	Adrenergic signaling in cardiomyocytes	BCL2, ADRB1, ADRB2, SCN5A, ADRA1A
hsa04917	Prolactin signaling pathway	GSK3B, MAPK8, ESR1, ESR2

### 3.7. Molecular docking analysis

**Figure 7.**
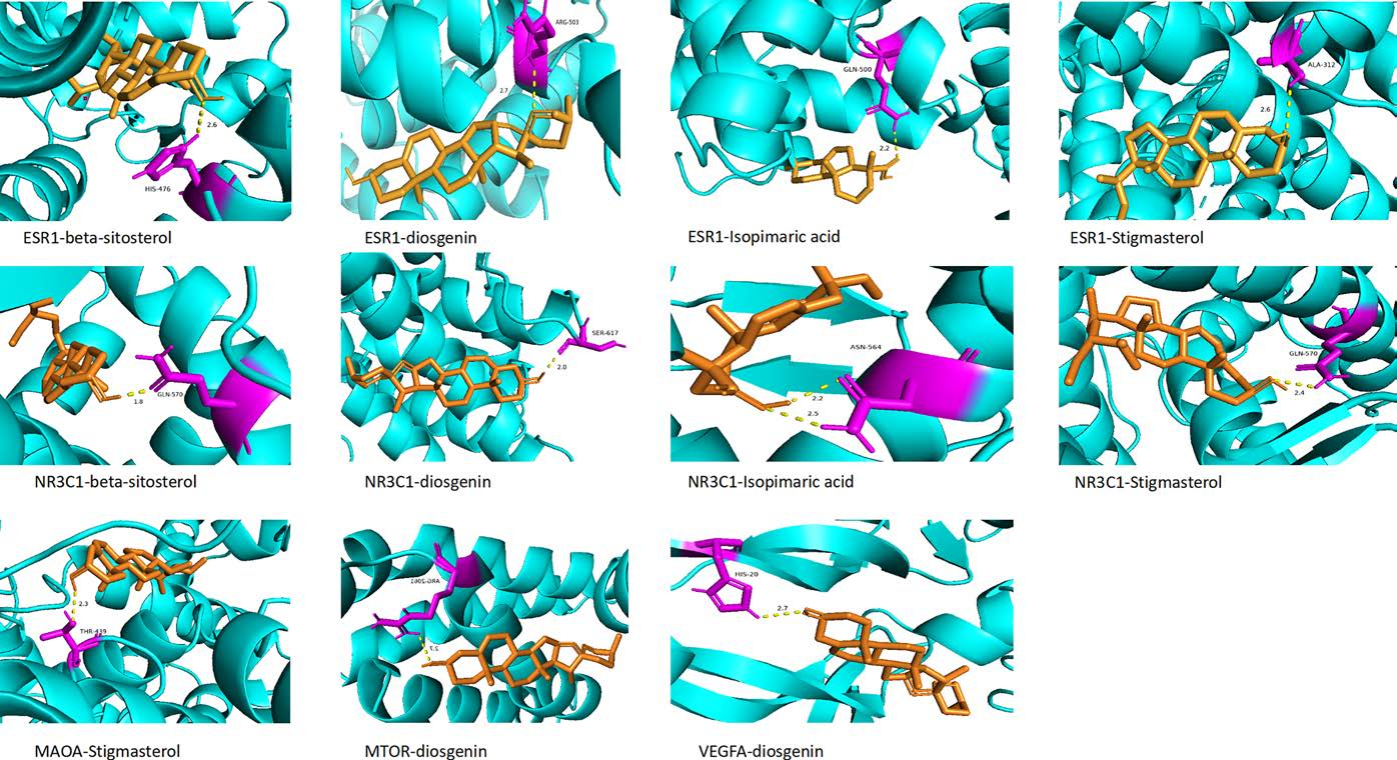
Molecular docking diagrams of PPD related targets with main compounds of BZD.

According to the five related signaling pathways from the results of KEGG pathway enrichment analysis, we identified five key genes: ESR1, MAOA, NR3C1, VEGFA, and MTOR. These key genes were mainly bound to diosgenin, isopimaric acid, stigmasterol, and beta-sitosterol, which are the main components of BZDs (Table 4). The molecular docking method verified the binding sites of BZD target genes and their corresponding compounds, showing that the above five target genes have a high affinity for the main components of BZD (Fig. 7).

**Table 4:** Results of the molecular docking of the five core genes with compounds of BZD.

Number	Core genes	Compound	Docking affinity
1	ESR1	Diosgenin	-9
2	ESR1	Isopimaric acid	-6.6
3	ESR1	Stigmasterol	-7
4	ESR1	beta-Sitosterol	-6.9
5	MAOA	Stigmasterol	-6.9
6	NR3C1	Isopimaric acid	-9.1
7	NR3C1	Diosgenin	-7.6
8	NR3C1	Stigmasterol	-8.6
9	NR3C1	beta-Sitosterol	-9.6
10	VEGFA	Diosgenin	-8.1
11	MTOR	Diosgenin	-7.5

## 4. Discussion

PPD is a common and serious mental health problem that causes personal suffering and interferes with parenting. However, the effects of antidepressant medications remain controversial. In the meantime, numerous people worry about the potential adverse effects of antidepressant medications. TCM has been used to treat mental illness in China for thousands of years. Therefore, the application of Chinese medicine can help develop new ideas and methods for the treatment of PPD.

Owing to the multi-target treatment effects of TCM, it can serve as a significant repository to develop drugs for the treatment of PPD. This study utilized network pharmacology and molecular docking simulations to reveal the molecular mechanisms of BZD in PPD treatment. BZD plays a potential role in treating PPD by regulating multiple target genes and pathways.

For this study, we selected 22 main compounds of BZD, among which icariin, timosaponin B-III (TB-III), and others are shown to have antidepressant effects. The potential target genes are *ESR1*, *MAOA*, *NR3C1*, *VEGFA*, and *mTOR*. Moreover, GO annotation and KEGG pathway enrichment analyses confirmed that these target genes were associated with HIF-1, dopaminergic synapse, estrogen, and serotonergic synapse signaling pathways, which are closely involved in the treatment of PPD. Molecular docking showed that the five core targets had a certain affinity for the main compounds of BZD.

TB-III is a steroidal saponin isolated from the rhizome of Baihe, which exhibits antidepressant activity through the regulation of inflammatory cytokines, BDNF signaling, and synaptic plasticity. However, this study was only conducted in a mouse model of PPD, and there have been no human reports.^[39]^ Icariin has potential preventive and therapeutic effects in various neurological diseases such as cerebral ischemia, depression, Parkinson’s disease (PD), and multiple sclerosis (MS). The mechanism by which icariin improves depression may be related to the promotion of cell proliferation, peripheral nerve regeneration, improvement of the function of damaged nerve regulation, decrease in glucocorticoid receptors (GRs) and 5-hydroxytryptamine 1A (5-HTR1A) receptors in the hippocampus and prefrontal cortex, regulation of the central neuroendocrine system, or restoration of the negative feedback regulation of the hypothalamic-pituitary-adrenal (HPA) axis.^[40–42]^ Furthermore, icariin may ameliorate prenatal restraint stress-induced depression-like behavior.^[43]^

The network pharmacology results confirmed that the potential target genes of PPD regulated by BZD were mainly *ESR1*, *MAOA*, *NR3C1*, *VEGFA*, and *mTOR*. Simultaneously, the PPI network results showed that these targets had close interactions. *ESR1* plays an important role in mediating hormonal differences during pregnancy and postpartum. One clinical experiment suggested a role for *ESR1* in the etiology of PPD, possibly through modulation of serotonin signaling.^[44]^ Previous work has demonstrated that exposure to and withdrawal from normal levels of gonadal steroids results in depressive symptomatology in women previously diagnosed with PPD.^[45,46]^ The *MAOA* gene, located on the short arm of the X-chromosome (Xp11.4-p11.3), has been the focus of research in the field of mental disorders in recent years. Two studies showed that *MAOA* was positive at 6 weeks postpartum with PPD.^[47,48]^ Women with PPD appear to have an abnormal HPA axis response to stress, which may involve genetic variants, as reported previously. The *NR3C1* gene, which is located on the long arm of chromosome 5 (5q31), encodes the GR. Epigenetic studies have shown that decreased *NR3C1* gene expression activity caused by methylation can impair the negative feedback regulation of the HPA axis, alter an individual’s response to SLE, and induce depression.^[49]^ However, the mechanisms of interaction between these targets are not clear. Other targets have not been directly documented to improve PPD, but the literature supports an improvement in depression, which follows the same pathway as PPD. For example, VEGFA can affect the complex processes of learning and memory^[50]^ and plays a role in regulating neurite growth and maturation during brain development.^[51]^ The role of VEGFA in neurogenesis may be mediated by its interactions with downstream effector genes.^[52]^ In the present study, our data showed that VEGF mRNA and protein expression in hippocampal tissues and serum were downregulated in depression model rats, suggesting that downregulation of VEGFA plays a key role in depression in rats. mTOR is a serine/threonine kinase that controls related signalling pathways to regulate many integrated physiological functions of the nervous system. Many studies have shown that it is tempting to hypothesize that the activation of mTOR function followed by enhanced mTOR-dependent protein synthesis may underlie the action of antidepressants, such as ketamine.^[53–56]^

GO annotation and pathway enrichment analyses were conducted to identify the potential biological functions of PPD targets. GO enrichment analysis revealed that the major biological processes included response to drugs, oxidation-reduction processes, response to lipopolysaccharide, response to estradiol, and response to hypoxia. The results of the KEGG enrichment analysis showed that HIF-1, dopaminergic synapse, estrogen, and serotonergic synapse signaling pathways were the leading signaling pathways for the treatment of PPD by BZD. These data suggest that the HIF-1 pathway might play an important role in antidepressant effects, and that altered mRNA expression of HIF-1 and its target genes in peripheral blood cells is associated mainly in a state-dependent manner with mood disorders, especially major depressive disorder (MDD).^[57]^ The estrogen signaling pathway plays an important role in PPD. Studies have shown that changes in gonadal steroid levels, including estrogen levels, may contribute to the depressive symptoms of PPD.^[58]^ Other data suggest that PPD symptoms are affected by estrogen levels.^[59,60]^ Serotonin regulates several basic biological functions relevant to depression, including sleep and appetite.^[61]^ Women with PPD have lower plasma serotonin levels than non-depressed controls, which are modulated by estrogen.^[62]^ Thus, fluctuating estradiol levels during pregnancy and the postpartum period may cause depressive symptoms in vulnerable women by destabilizing the serotonin system. Dopamine is recognized and proven to be an important factor, and diminished dopaminergic function may also play a role in PPD. Studies have shown that dopamine activity and estradiol levels are positively correlated; therefore, they can synergistically affect PPD symptoms.^[63]^

Molecular docking verified that ESR1, MAOA, NR3C1, VEGFA, and mTOR have high affinities for the main active ingredients of BZD, diosgenin, isopimaric acid, stigmasterol, and beta-sitosterol, providing data support for BZD as a potential drug for the treatment of PD; however, further experimental studies are needed to confirm its effectiveness.

## 5. Conclusion

In this study, we used network pharmacology combined with molecular docking to elucidate the mechanism by which BZD regulates PPD through multiple targets and channels. The main target proteins of BZD in PPD treatment have been shown, constructing a target protein network. BZD mainly affected HIF-1, dopaminergic synapse, estrogen, and serotonergic synapse signaling pathways while regulating the key target proteins of ESR1, MAOA, NR3C1, VEGFA, and mTOR.

However, this article also has some limitations. The composition of traditional Chinese medicine is complex, the data of this article is obtained through some databases, and the information of the databases needs to be further improved. The validity of this article lacks experimental support, and its mechanism needs further experimental verification.

## Author contributions

Qiong Zhao, Wengu Pan, and Guomin Si performed the main analyses and drafted the manuscript. Hongshuo Shi and Fanghua Qi designed the study. Yuan Liu helped with the introduction and discussion. Tiantian Yang and Hao Si assisted in the preparation of the manuscript. All authors wrote, read, and approved the manuscript.

Conceptualization: Guomin Si.

Data curation: Hongshuo Shi, Fanghua Qi and Hao Si.

Formal analysis: Qiong Zhao, Wengu Pan, Yuan Liu, Tiantian Yang.

Funding acquisition: Qiong Zhao, Wengu Pan.

Investigation: Qiong Zhao, Wengu Pan, Guomin Si, Fanghua Qi.

Methodology: Qiong Zhao, Guomin Si, Tiantian Yang.

Project administration: Guomin Si.

Resources: Qiong Zhao, Wengu Pan, Hongshuo Shi, Fanghua Qi, Yuan Liu, Tiantian Yang, and Hao Si

Software: Qiong Zhao, Hao Si.

Supervision: Guomin Si.

Validation: Guomin Si, Wengu Pan, Hongshuo Shi.

Visualization: Wengu Pan, Hongshuo Shi.

Writing – original draft: Qiong Zhao, Wengu Pan, Guomin Si, Fanghua Qi.

Writing – review & editing: Qiong Zhao, Guomin Si.
